# Fellowship for Women Physicians

**Published:** 1913-03

**Authors:** 


Fellowship for Women Physicians. The Mary Putnam Jacobi fellowship of $800 is open to all women graduates, selection being made by examination of credentials and deciding as to who is most apt to prove most worthy of assistance in post-graduate studies, to be pursued in New York City. The award will be made June 1, 1913, to cover the year beginning October 1, 1913. Applications should be made to Dr. Emily Lewi, 35 Mt. Morris Park, West, New York City.

